# Portals to Wonderland: Health portals lead to confusing information about the effects of health care

**DOI:** 10.1186/1472-6947-5-7

**Published:** 2005-03-15

**Authors:** Claire Glenton, Elizabeth J Paulsen, Andrew D Oxman

**Affiliations:** 1 Informed Choice Research Department, Norwegian Health Services Research Centre, Pb. 7004 St. Olavs Plass, 0130 Oslo, Norway

## Abstract

**Background:**

The Internet offers a seemingly endless amount of health information of varying quality. Health portals, which provide entry points to quality-controlled collections of websites, have been hailed as a solution to this problem. The objective of this study is to assess the extent to which government-run health portals provide access to relevant, valid and understandable information about the effects of health care.

**Methods:**

We selected eight clinically relevant questions for which there was a systematic review, searched four portals for answers, and compared the answers we found to the results of the systematic reviews.

**Results:**

Our searches resulted in 3400 hits, 155 of which mentioned both the condition and the intervention in one of the eight questions. Sixty-three of the 155 web pages did not give any information about the effect of the intervention. Seventy-seven qualitatively described the effects of the intervention. Twenty-six of these had information that was too unclear to be categorised; 15 were not consistent with the systematic review; and 36 were consistent with the review, but usually did not mention what happens without the intervention, what outcomes have been measured or when they were measured. Fifteen web pages quantitatively described effects. Four of these were abstracts from the systematic review, nine had information that was incomplete and potentially misleading because of a lack of information about people not receiving the intervention and the length of follow-up; one had information that was consistent with the review, but only referred to three trials whereas the review included six; and one was consistent with the review.

**Conclusion:**

Information accessible through health portals is unlikely to be based on systematic reviews and is often unclear, incomplete and misleading. Portals are only as good as the websites they lead to. Investments in national health portals are unlikely to benefit consumers without investments in the production and maintenance of relevant, valid and understandable information to which the portals lead.

## Background

*"'Be what you would seem to be' – or, if you'd like it put more simply – 'Never imagine yourself not to be otherwise than what it might appear to others that what you were or might have been was not otherwise than what you had been would have appeared to them to be otherwise."' *(Lewis Caroll (1832 – 1898), Alice in Wonderland)

The internet offers a seemingly endless amount of health information, but the quality of this information is variable [1]. A bewildering array of criteria are used to rate the quality of this information, but none of these have been validated and many of them have a short life span [2]. Guidelines exist to help consumers to critically appraise the relevance and validity of health information [3-6], but few people have the time and skills to apply such guidelines.

Health portals, which provide entry points to quality-controlled collections of websites, have been hailed as a solution to these problems. The development of health portals by national governments can be seen as a support of recent legislation and policies establishing the right for individuals to participate in decisions regarding their health care. Information that supports an informed choice about health care should include reliable information about the relative benefits and harms of relevant options [7]. Moreover, this information should be presented in such a way that it is easily understood. Consistent presentations across various treatment options can make it easier to understand information and makes it easier to make comparisons across treatments.

The objectives of this study were to assess the extent to which health portals provide easy access to relevant, valid and understandable information about the effects of health care. We examined four English-language government-run health portals:

• Canadian Health Network – Canada [8]

• HealthInsite – Australia [9]

• MEDLINEplus – USA [10]

• NHS Direct Online – England [11]

These four portals lead to similar types of resources including information about health conditions and treatments. They describe their goal as the provision of "*appropriate*", "*authoritative*", "*credible*" and "*timely*" health information for the general public. Table 1 summarises their guidelines for including sites.

**Table 1:** Health portal goals and selection guidelines

**Portal**	**Goal**	**Selection guidelines for included sites**
**Canadian Health Network**	"CHN's mission is to support Canadians in making informed choices about their health, by providing access to multiple sources of credible and practical e-health information."	• Does the organization have timely and credible information on health promotion and disease prevention? • Is authorship clearly stated? • If the source is not a professionally or legally accredited authority, is there sufficient supporting information to establish an informed perspective. • If a non-professional gives medical information, is this fact clearly stated? • Are original sources clearly referenced? • Are claims relating to the benefits of a specific health promotion/disease prevention services supported by appropriate, balanced evidence? • Is the content original, and of sufficient depth to warrant a link? • Is the resource relevant to the CHN mission statement? • Is the information updated or reviewed on an adequate basis given the content? • If the resource only presents one-side of a controversial issue, is this made explicit?

**HealthInsite**	"(Health*Insite*) aims to improve the health of Australians by providing easy access to quality information about human health."	Sites must have a written policy and procedure that • Includes a policy that each resource is authored by a person or group with appropriate qualifications/experience • Includes a procedure for appropriate attribution of resources • Includes a review process. The policy needs to cite positions/qualifications/names of who reviews • Details the final approval process (including responsibility/qualifications) • The date of publication and, where appropriate, date of previous versions must also be given.

**Medline Plus**	"MedlinePlus is designed to help you find appropriate, authoritative health information."	• The source of the content is established, respected and dependable. A list of advisory board members or consultants is published on the site. • The information provided is appropriate to the audience level, well-organized and easy to use. • Information is from primary resources (i.e., textual material, abstracts, Web pages). • The primary purpose of the Web page is educational and not to sell a product or service. • The source for the contents of the Web page(s) and the entity responsible for maintaining the Web site is clear. • Information is current or an update date is included. • The site provides unique information to the topic with a minimum of redundancy and overlap between resources.

**NHS Direct Online**	To provide high quality health information and advice for the people of England and Wales	No information provided.

We used each portal to find information about the effects of interventions for eight health problems (table 2) and compared the information we found to the results of systematic reviews [12-20].

**Table 2:** Health care questions

Problems	Interventions	Reference
Alzheimer's disease	Galantamine	8
Bulimia	Antidepressants versus psychological treatment versus both	9
Jet lag	Melatonin	10
Lumbar disc prolapse	Surgery	11
Malaria prevention	Mefloquine	12
Nausea and vomiting in early pregnancy	Treatments	13,14*
Schizophrenia	Haloperidol	15
Vaginal yeast infection	Oral versus intravaginal antifungal agents	16

*A substantive update of this review was made in 2004. This was taken into account when comparing website information with the results of the review.

## Methods

Research topics covered by systematic reviews were identified by searching through the journals *Evidence Based Mental Health *and *Evidence Based Medicine *2000–2002. Topics presented in these journals are selected for their presumed clinical relevance. For the sake of consistency, we chose reviews from The Cochrane Library. Topics were chosen to cover a variation in age, sex, mental/physical health, and chronic/acute illness.

Search terms for the health care conditions and interventions were developed (See table 3). Search terms that lay people were likely to use were preferred, and were also adapted to local spellings and expressions. Where there was a choice between several search terms, the term that gave the most hits was chosen. We also used the portals' lists of health topics and indexes to search for relevant information.

**Table 3:** Search terms

Alzheimer's disease	alzheimer +alzheimer +galantamine alzheimer and galantamine/reminyl galantamine alzheimer alzheimer galantamine/reminyl galantamine/galanthamine reminyl
Bulimia	+bulimia +treatment bulimia and treatment bulimia treatment bulimia

Jet lag	jeg lag and melatonin jet lag melatonin jet lag melatonin

Lumbar disc prolapse	lumbar disc prolapse herniated disk/disc AND surgery herniated disc/disk slipped disc/disk discectomy

Malaria prevention	mefloquine and malaria mefloquine malaria mefloquine malaria

Nausea and vomiting in early pregnancy	morning sickness morning sickness and treatment pregnancy nausea early pregnancy nausea

Schizophrenia	+haloperidol +schizophrenia schizophrenia and haloperidol schizophrenia haloperidol haloperidol schizophrenia

Vaginal yeast infection	+yeast +infection +treatment +thrush +treatment yeast infection and treatment yeast infection treatment yeast infection thrush candidiasis

Between January and February 2004 two researchers (CG and EJP) independently carried out searches for each topic within each portal. Web pages were included if they referred to both the condition and the intervention. When the systematic review in question covered several interventions, web pages describing any one of these interventions were included. Web pages were only included if the portal linked directly to them or to other pages on the same web site.

Web pages were excluded if they were duplicates or if they were clearly not intended as information about the conditions and interventions. Information intended for a professional audience was also excluded when a consumer version of the same information was available. Discrepancies between the researchers' search results were resolved through discussion.

Included web pages were then examined to see if they included either qualitative or quantitative information about the effects of the interventions. Qualitative information was categorised as indicating an effect, a probable effect, an unknown effect, probably no effect, no effect, or unclear, if it was not possible to categorise the information (Table 5). The results of the systematic reviews were categorised using the same categories to allow comparison of the qualitative information we found to the results of the reviews. Quantitative information was compared directly to the results of the reviews.

**Table 5:** Coding of qualitative information

Statements coded as expressing effect	*You may benefit from the medication* *It has helped many patients* *It is said to improve symptoms*
Statements coded as not expressing effect	*The treatment can be used* *Treatment may include* *Your doctor may recommend the treatment*

Statements coded as "effective"	*Is effective/highly effective/(often) very effective* *Has proven effective/helpful/beneficial* *Especially helpful* *Has been found/reported to be helpful/effective* *Has an effect on the symptoms* *Improves the symptoms* *Enjoys a good success rate* *Works/(usually) works well* *Usually successful* *Is the most effective drug* *X was more effective than Y* *Was better than other therapies and better than no treatment* *Strong evidence on the effectiveness of X when compared with Y* *In clinical trials, some people who took the drug compared to individuals who took a placebo showed some improvement* *People treated with the drug showed improvements in symptoms* *Reduces the PANSS scores significantly more than placebo* *You can protect yourself against X by taking Y* *Can clear up your symptoms in a couple of days and cure the infection within a week* *Many studies have shown the treatment to be useful/can ease the symptoms* *Many people respond to the treatment* *Most people do very well after treatment* *These treatments have been beneficial to many women* *Relieves symptoms in the majority of properly selected patients* *Have helped many patients* *Is recommended/should be recommended* *These drugs all had similar ability to relieve the symptoms* *Helps some sufferers/some people* *Of some value in certain patients*

Statements coded as "probably effective"	*There have been reports of some benefits* *Appears to be helpful* *Results/evidence suggest that the treatment is beneficial/has beneficial effects*

Statements coded as "unknown effect"	*Have not been proven* *Results are variable* *Some studies suggest this is effective..other studies have found that it doesn't help* *Effectiveness for many people is unknown* *Results of studies have been inconsistent* *The evidence is uncertain* *The treatment is at present, an experimental approach*.

Statements coded as "not effective"	None found

Statements coded as "probably not effective"	None found

Statements coded as unclear	*Not all people taking these drugs benefit from them* *The treatment is not always successful* *For some people, the treatment may help prevent some symptoms from becoming worse* *For some people, the treatment is prescribed to possibly delay the worsening of some of their symptoms* *Does the treatment work? Not always* *The treatment is of not more benefit than the other treatments* *May help/relieve pain/improve symptoms/relieve symptoms/be a good option* *Might help* *Some studies have shown that (the treatment) may decrease (the symptoms)* *Can help/improve symptoms/relieve symptoms* *Is said to improve symptoms* *The effects of the treatment varies for different people. Some will not notice any effect, while others may find that their condition improves slightly*

## Results

**Figure 1:**
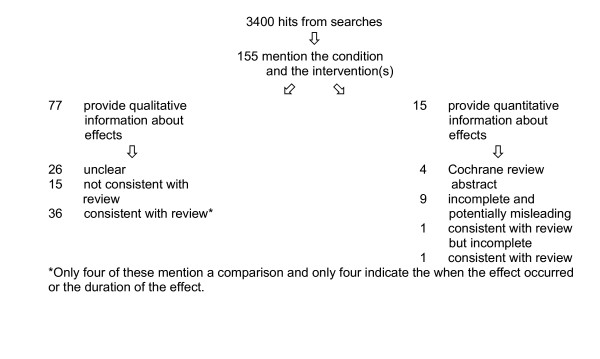
Flowchart showing the number of web pages identified for each category.

Our searches resulted in roughly 3400 hits. Searching was sometimes made difficult by the variety of terms used for the same conditions and interventions. For instance, the use of different terms for vaginal yeast infection, for the same groups of anti-psychotics, and for similar types of back surgery was confusing and resulted in different numbers of hits within each portal. Searches were possibly more extensive than necessary as our goal was to identify as much relevant information as possible, rather than to test the sensitivity and specificity of the search engines. After removing duplicates and excluding web pages that were clearly not relevant, (for instance, minutes of meetings or reports on cost-effectiveness) a total of 155 web pages that mentioned both the condition and the intervention(s) for one of the eight questions remained (figure 1). All four portals provided information about each of the eight questions.

Sixty-three of the 155 web pages that mentioned both the condition and the intervention in question did not give any information about the effect of the intervention. The remaining 92 web pages presented information about effect either in qualitative or quantitative terms.

### Qualitative information

Seventy-seven of the included web pages qualitatively described the effects of the interventions. It was frequently difficult to judge what was and was not a statement about the effects of an intervention and what a statement meant in terms of the effectiveness of an intervention. We therefore asked 16 colleagues to categorise a selection of statements. Frequently there was little agreement among them. The use of the word "may" was particularly confusing. For instance, the statement "for some people in the early and middle stages of the disease, galantamine may help prevent some symptoms from becoming worse for a limited time" was categorised as "effective" by one person, "probably effective" by seven, "unknown effectiveness" by five, and "unclear" by three. Statements about what clinicians do were also confusing, for example "your doctor may recommend surgery."

Based on the input of our colleagues the three of us agreed on the final code list (Table 5) that was used to categorize the qualitative statements of effect. The specific context of a statement was also taken into account in the coding.

Twenty-six of the 77 web pages with qualitative statements of effect were categorised as "unclear" because it was not possible to determine what the statement meant about the effectiveness of the intervention. Of the 51 web pages that could be categorised 15 were not consistent with the results of the systematic review and 36 were consistent. However, only four of the 36 that were consistent described what happened to people who did not receive the intervention and only four indicated how long the effect was likely to last.

In some cases, portals offered contradictory information. While websites on Health Insite, Canadian Health Network, and NHS Direct Online all support the use of ginger for the treatment of morning sickness, websites on Medline Plus include the following messages:

• *"Try using ginger, which has proved effective in combating morning sickness."*

• *"Ginger has been studied in pregnant women and appears to be helpful."*

• *"Ginger" is not recommended for morning sickness, especially if you have a history of bleeding disorders or miscarriage. It may cause bleeding or impair fetal development*.

### Quantitative information

Fifteen of the 155 included web pages quantitatively described the effects of the intervention. Some of these web pages also gave qualitative information, but in these cases only the quantitative information was categorised. Effects were presented in a variety of ways, including percentages, numbers needed to treat, frequencies and fractions. Four of the 15 web pages were abstracts of Cochrane systematic reviews. Nine had information that was judged to be incomplete and potentially misleading because outcomes for people who did not receive the intervention was not reported. In eight of these nine web pages, the length of follow-up was also not reported, despite the fact that this was relevant to the conditions in question. For example, one web page states that "good results are achieved in 80% to 90% of the cases" for patients receiving surgery for lumbar disc prolapse, implying that 80 to 90% of patients benefit from surgery, without specifying what proportion of similar patients have good results without surgery, what outcome measures "good results" refer to, or how long after surgery. The systematic review found that discectomy appeared to give fast short-term pain relief, but there was no good estimate of this effect, and that the effect on long-term pain relief was unknown.

Two of the fifteen web pages were consistent with the systematic review. One of these web pages compared the effect of oral versus vaginal antifungals for yeast infection. The other web page reported the effect of galantamine for Alzheimer's disease and was the only one, besides the Cochrane systematic review abstracts, that provided information about outcomes for people not using the intervention. This information, however, referred to three trials, whereas the systematic review included six trials.

## Discussion

The goal of this study was to evaluate the reliability of information about the effects of health care accessed through health portals that aim to facilitate access to reliable information. In most cases, it was difficult to know whether the information was reliable, in comparison to the results of systematic reviews, because of the incompleteness and vagueness of the information.

Generally, websites to which the portals led us did not compare the effects of an intervention with no intervention or an alternative intervention. They also did not give any information about when outcomes were measured or what outcomes were measured in the studies on which the information was based. The evidence on which the information was based was usually not stated.

Most of the web pages with information about effectiveness described effects qualitatively. This may reflect an assumption that qualitative presentations are easier to understand, or that information providers do not wish to express more than they know [21]. However, qualitative descriptions mean different things to different people [21-23]. The difficulties that we and our colleagues had in categorising qualitative descriptions of effects is indicative of the difficulties that others are likely to have understanding the information that the portals lead to. Although most of the information was not as confusing as the quote from Alice in Wonderland at the beginning of this article, it was confusing and often left us wondering what the intended message was and how it related to the available evidence.

Quantitative presentations are more precise and less open to misinterpretation, but may be regarded as being more difficult to understand [21], and may be open to manipulation through framing [24]. The abstracts from Cochrane systematic reviews, which were included in web pages to which two of the portals led (table 4), frequently use research jargon and are likely to be inaccessible for many consumers [7]. Nine of the 11 other web pages we included that presented quantitative information presented incomplete information.

**Table 4:** Included web pages per portal

**Portal**	**Included hits**	**Information about effects**	**Qualitative statements about the effect of the intervention**				**Quantitative statements about the effect of the intervention**				
			
			*Qualitative *statement	Statement unclear	Statement not consistent with review	Statement consistent with review	*Quantitative *statement	Cochrane review abstract	Statement incomplete and potentially misleading	Statement consistent but incomplete	Statement consistent with review
**Canadian Health Network**	22	13	12	2	2	8	1	0	1	0	0
**Health-Insite**	38	21	15	5	2	8	6	3	2	1	0
**Medline Plus**	77	46	41	17	9	15	5	0	4	0	1
**NHS Direct Online**	18	12	9	2	2	5	3	1	2	0	0
**TOTAL**	**155**	**92**	**77**	**26**	**15**	36*	**15**	**4**	**9**	**1**	**1**

*Only four of the 36 described what happened to people who did not receive the intervention and only four indicated how long the effect was likely to last.

None of the health portals report considering how effects are presented in their selection criteria. Included web pages present information about the effects of health care in a variety of ways. Most often this information is presented in ways that may confuse and mislead users. The nature of portals limits any possibility of addressing these problems or providing user support for understanding the effects of health care.

By collecting websites from numerous sources, governments can avoid "recreating existing health information" and are able to present information "from multiple perspectives" [8]. However, to compare the benefits of alternative interventions, it is still necessary for users to search through numerous web sites and piece together information that frequently uses different terminology for the same interventions and conditions, is presented in different formats, and is often vague, incomplete and difficult to understand. Portals depend on having information to which they can link. If reliable and understandable information does not already exist, the portals are of little value.

## Conclusion

The information accessible through these four portals displays the same weaknesses as patient information generally [1,25]. It is seldom based on systematic reviews and is often unclear, incomplete and misleading. People going through these portals to find information that can help them make an informed choice about any of the questions we posed are likely be disappointed with what they find. Portals are only as good as the websites they lead to. Establishing and maintaining national health portals is only worth the investment if an investment is also made in producing and maintaining relevant, valid and understandable information to which the portals lead.

## Competing interests

All three of the authors contribute to the Cochrane Collaboration. CG and AO contribute to the development of Internet-based patient information.

## Authors' contributions

CG and AO conceived the idea for this study. CG and EP developed the protocol with assistance from AO, collected and analyzed the data. CG drafted the manuscript. All three authors contributed to the final manuscript. CG and EP will act as guarantors of the paper.

## Pre-publication history

The pre-publication history for this paper can be accessed here:


